# Role of the frontal aslant tract in language preservation and recovery after surgery: a multicenter analysis of patients with left frontal glioma

**DOI:** 10.3389/fneur.2026.1855849

**Published:** 2026-07-03

**Authors:** L. F. Salvati, R. De Marco, F. Balletti, A. Morello, A. Gatto, A. Leocata, P. Fiaschi, S. Caneva, B. Cagetti, M. Truffelli, F. Bruno, A. Tabano, G. Zona, F. Cofano, R. Rudà, D. Garbossa, A. Bianconi

**Affiliations:** 1 Division of Neurosurgery, ASST Sette Laghi, Varese, Italy; 2 Division of Neurosurgery, Santa Corona Hospital, Pietra Ligure, Italy; 3 Department of Neuroscience "Rita Levi Montalcini", University of Turin, Turin, Italy; 4 Neurosurgery Unit, University Hospital "Città della Salute e della Scienza", Turin, Italy; 5 Neurosurgery Unit, IRCCS Azienda Ospedaliera Metropolitana, Genoa, Italy; 6 Department of Neuroscience, Rehabilitation, Ophthalmology, Genetics, Maternal and Child Health (DINOGMI), University of Genoa, Genoa, Italy; 7 Department of Clinical Psychology and Psychotherapy, IRCCS Policlinico San Martino, Genoa, Italy; 8 Division of Neuro-Oncology, Department of Neuroscience, University and City of Health and Science Hospital, Turin, Italy

**Keywords:** diffusion tensor imaging, fat, frontal aslant tract, glioblastoma, glioma, language, tractography

## Abstract

**Introduction:**

In glioma surgery, achieving maximal safe resection while preserving neurological function can be highly challenging, and the knowledge of white matter connectivity plays a key role in achieving this goal. Regarding language functions, the frontal aslant tract (FAT) plays a unique role in coordinating verbal sequences in the dominant hemisphere. Damage to this tract is associated with the development of verbal fluency disorders of varying severity and duration; however, its significance in the language function, as well as the association between the extent of damage and functional recovery, has not yet been well established.

**Methods:**

This prospective multicenter study, conducted at three different centers in northern Italy, aimed to evaluate the involvement of the FAT in the language function by analyzing a cohort of adult patients undergoing surgery for glial lesions in the dominant hemisphere and following them for 3 months to assess the potential for function recovery. Preoperative magnetic resonance imaging (MRI), with diffusion-weighted imaging (DWI) and diffusion tensor imaging (DTI) sequences—performed using deterministic algorithms implemented in Brainlab iPlan software—was compared with postoperative imaging to determine the extent of FAT injury.

**Results:**

A total of 51 patients who underwent tumor excision surgery were recruited for the study, with maximal tumor resection achieved in 58.8% of cases. Language function worsened postoperatively in 29 patients (56.9%), with the onset or worsening of aphasia. At the 3-month follow-up, persistent language deficits were observed in 14 cases. Surgical manipulation of the FAT was strongly associated with the development of language impairment, particularly with respect to the volume of fibers resected and the segment of FAT involved. Resection of the middle segment was associated with both immediate postoperative language worsening and poor long-term language outcomes. The integrity of this segment was also associated with the possibility of neurological recovery over time. A FAT resection volume of at least 0.5 cm^3^ was found to be a significant predictor of persistent language deficits.

**Discussion:**

Knowledge derived from tractography may help guide safe glial tumor resection while preserving language function, even when FAT fibers are involved. Since language deficit may be irreversible, particular attention should be paid to the middle segment and the volume of FAT fibers intersected intraoperatively.

## 1 Introduction

In the management of gliomas, despite advancements in adjuvant therapies, surgery remains a cornerstone, playing a pivotal role in overall patient survival as well as in oncological and functional outcomes (1–3). Achieving maximal safe resection while preserving neurological function can be highly challenging and requires a thorough knowledge of cortical areas and of the brain’s connectome framework (4). The importance of white matter connectivity in guiding surgical strategies has led to the development of non-invasive preoperative techniques for studying the connectome, including diffusion tensor imaging (DTI) tractography, functional MRI, and navigated repetitive transcranial magnetic stimulation (nTMS) (5–7). These techniques play a key role in language functions, particularly when direct mapping during awake surgery cannot be achieved (8, 9).

Human language processing has been well described according to the dual-stream model; however, a role in multiple aspects of language processing has also been attributed to a more recently characterized white matter bundle, the frontal aslant tract (FAT) (10–12). Although the hemispheric lateralization of FAT remains unclear, evidence suggests that the left hemisphere fibers play a role in language production, verbal planning, and control of the articulatory apparatus (13–15). Injury to the FAT may result in aphasia, speech arrest or delayed speech initiation, reduced verbal fluency, errors in verb generation tasks, and impairment in reading and simple calculations (14, 16). Data on the functional consequences of surgical injury to white matter tracts remain limited. Although FAT damage related to tumorous infiltration or surgical injury does not seem to be associated with permanent aphasia, full and rapid functional recovery is possible; however, no clear association has been found between the extent of FAT damage and functional recovery (17). Some authors have proposed dividing the FAT into three segments, and studies on FAT neuroplasticity have demonstrated an anterior-to-posterior gradient, with a higher compensatory potential in the anterior and middle segments, while the posterior (motor) segment shows limited capacity for compensation following injury (18–20).

This study aimed to investigate the role and functional relevance of the FAT in language function by analyzing surgery-related deficits in patients undergoing left frontal glioma surgery in the dominant hemisphere. The main outcomes of the study were the severity of the deficit related to the FAT region involved and the duration of symptoms, with the aim of assessing the potential development of compensatory mechanisms or network plasticity associated with functional recovery during a 3-month follow-up period.

## 2 Methods

### 2.1 Study design and setting

This multicenter study was conducted between August 2020 and December 2025 at three different centers in northern Italy: the “Città della Salute e della Scienza-Molinette,” University Hospital in Turin; the “IRCCS Policlinico Ospedale San Martino,” University Hospital in Genoa; and the “Ospedale Santa Corona,” Hospital in Pietra Ligure. The following inclusion criteria were adopted: adult patients (>18 years old); native Italian speakers; patients affected by intra-axial lesions in the frontal lobe of the left hemisphere; lesions with radiological features suggestive of low-grade gliomas (LGGs) or high-grade gliomas (HGGs) identified through brain MRI with gadolinium and with available DWI and DTI sequences; and patients with histologically confirmed glial lesions, according to the 2021 WHO classification, following neurosurgical resection (21).

The study was conducted in accordance with the recommendations guiding physicians in biomedical research involving human subjects of the 1964 Declaration of Helsinki and its later amendments. Written informed consent was obtained from all patients for medical evaluation, treatment, and the use of their personal data for scientific purposes.

### 2.2 Clinical examination of language function and aphasia score

Demographic and clinical data were prospectively collected from medical records. Preoperative neurological presentation was described, with particular reference to alterations in language function. Patients underwent speech therapy assessments through the administration of the Italian version of the Aachen Aphasia Test (AAT), which consists of different subtests assessing distinct linguistic domains, including the Token Test, object naming, oral and written comprehension, and word repetition (22). Based on the test results, patients were classified according to the severity of aphasia into four groups: group 0 = no aphasia (≥90% AAT score); group 1 = mild aphasia (89–75% AAT score); group 2 = moderate aphasia (74–55% AAT score); and group 3 = severe aphasia (<55% AAT score). The AAT test was administered before surgery, within the first postoperative week, and during follow-up at 1 and 3 months after surgery. A comparison of all the results obtained helped determine whether new deficits occurred after surgery and whether language alterations improved, worsened, or remained stable over time, enabling the classification of aphasia as transient or permanent.

### 2.3 Image acquisition and analysis

**Figure 1:**
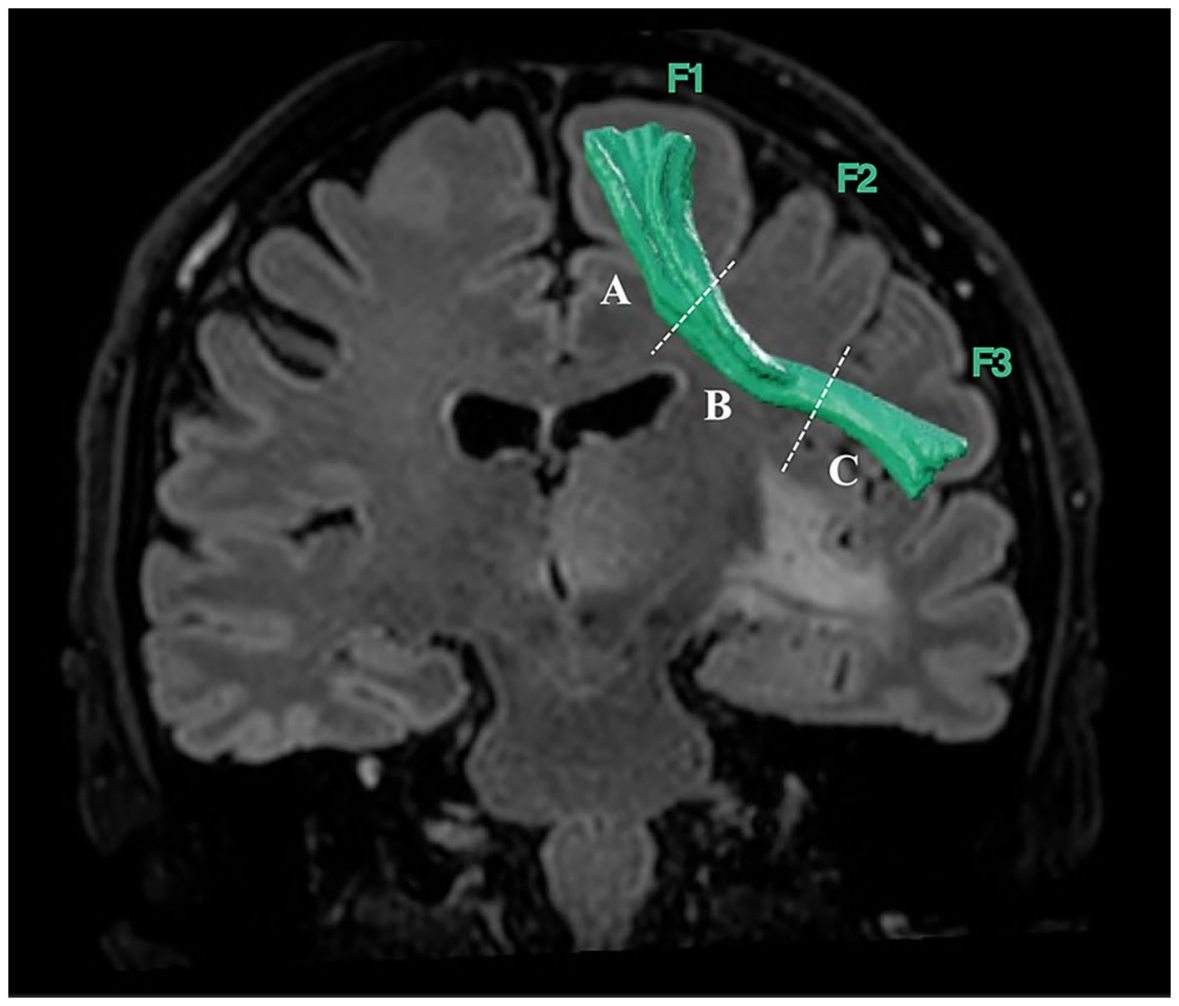
FAT segmentation: the medial segment **(A)** the superior frontal sulcus—the middle segment **(B)** the middle frontal sulcus—the lateral segment **(C)**.

During the preoperative assessment, each patient underwent contrast-enhanced brain MRI, including T1-weighted volumetric sequences, T2-weighted/fluid-attenuated inversion recovery (FLAIR) sequences, and diffusion-weighted sequences. T1-weighted gadolinium-enhanced sequences for high-grade gliomas (HGGs) and FLAIR sequences for low-grade gliomas (LGGs) were used to calculate tumor volume (expressed in cm^3^) using the semiautomatic segmentation tool available in Brainlab Elements (BrainLAB AG, Munich, Germany). The anatomic location of the lesion was recorded in terms of side and involved regions, including only tumors located in the frontal lobe of the left hemisphere. Acquisitions for tractography were performed for each examination using deterministic algorithms implemented in Brainlab iPlan software. Regions of interest (ROIs) were placed according to Fekonja et al. They were also validated in cases of displaced, disrupted, or infiltrated fiber tracts (20). Tumor-to-tract distance (TTD) was calculated, with the FAT involvement defined as a TTD of 0 mm. According to Tuncer et al. (19), the FAT has been subdivided into three segments: medial or superior (A), middle (B), and lateral or inferior (C) (Figure 1).

**Figure 2:**
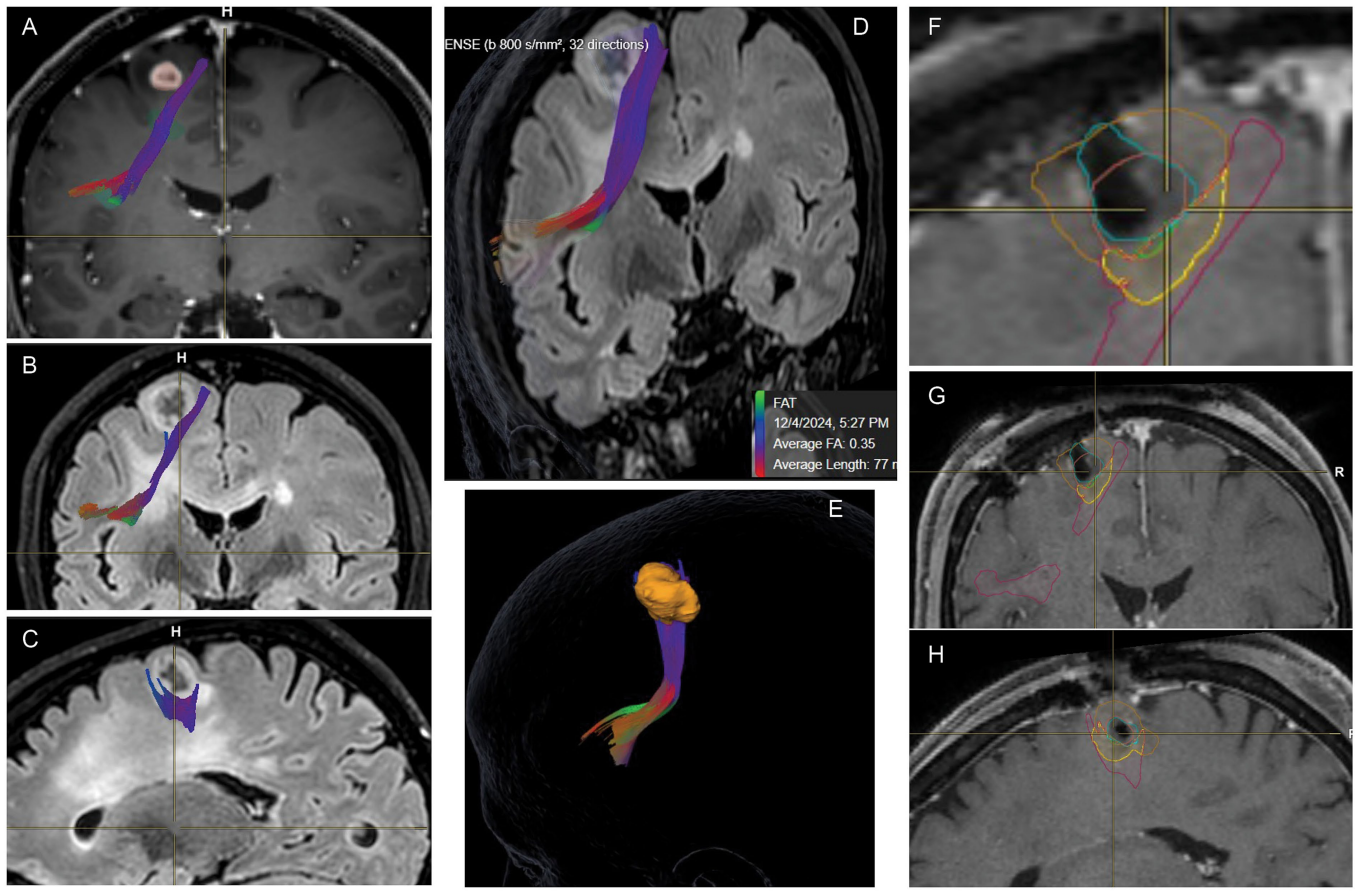
Preoperative and postoperative tractography studies. **(A)** Relationship between the tumor and FAT fibers; T1-weighted gadolinium-enhanced sequence, coronal view. **(B, D)** Relationship between the tumor and FAT fibers; FLAIR sequences, coronal view. **(C)** Relationship between the tumor and FAT fibers; FLAIR sequences, sagittal view. **(E)** Three-dimensional reconstruction showing the spatial relationship between the tumor (orange)and FAT, with the reconstructed bundle in purple/blue and the fiber endpoints color-coded in green and red according to fiber orientation. **(F**, **G)** Pre- versus post-operative comparison obtained by overlaying, on post-operative MR images the segmented volumes: tumor on pre-operative FLAIR sequence (orange), pre-operative FAT (magenta), portion of the FAT infiltrated by the tumor (yellow), post-operative resection cavity (cyan), and resected portion of the FAT (green); coronal view, with **(F)** magnified view and **(G)** overview. **(H)** Same overlay of pre- and post-operative volumes in sagittal view.

The association between the tumor and the white matter tracts was analyzed in terms of its intersection with the FAT fibers (Figure 2). The segments of the FAT involved were described, and the volume of intersection was studied (expressed in cm^3^). Tractography data were integrated into the intraoperative neuronavigation setting and were available during surgery to guide maximal safe tumor resection. When feasible, awake surgery with language mapping was performed. Postoperative contrast-enhanced brain MRI was performed within 72 h after surgery and at 1 month of follow-up. Subsequently, in the first postoperative MRI, T1-weighted gadolinium-enhanced sequences for HGGs and FLAIR sequences for LGGs were used to segment the surgical cavity to assess the presence of residual tumor volume. If present, it was compared with the preoperative volume to define the extent of resection (EOR) and whether a partial resection (PR) or subtotal resection (STR) was obtained; a gross total resection (GTR) was documented if no residual tumor was found. To analyze potential surgery-related FAT injury, preoperative MRIs with DTI sequences and postoperative MRIs were compared using Brainlab software. This comparison enabled the identification of the FAT segments involved during surgery and the estimation of the volume of its probable resection performed intraoperatively (expressed in cm^3^) (Figure 2).

### 2.4 Statistical analysis

Demographic, radiological, and surgical variables were analyzed: nominal or ordinal data were described using absolute and relative frequencies, while continuous variables were described using median and interquartile range (IQR) or mean and standard deviation (SD).

A univariate analysis was performed to assess the potential association between alterations in language, measured by AAT scores, and the proximity or overlap between the FAT and the surgical cavity. Normally distributed continuous variables were analyzed using an analysis of variance (ANOVA) linear model, non-normally distributed continuous variables using the Mann–Whitney *U*-test, and dichotomous and categorical variables using Pearson’s chi-squared test. Binomial logistic regression-based models were used to assess the relationship between independent variables and AAT scores at follow-up, with the extent of FAT surgical involvement during surgery considered as the dependent variable; adjusted odds ratios (ORs) and their 95% CIs were calculated.

## 3 Results

### 3.1 Patient and tumor characteristics

A total of 51 patients undergoing brain tumor surgery were analyzed. Of the 51 patients, 36 (70.6%) had IDH (isocitrate dehydrogenase)-wildtype and 15 (29.4%) had IDH-mutant gliomas. The mean preoperative tumor volume was 47.4 cm^3^ (SD 38.9, range 0.9–160.0), calculated using contrast-enhanced T1-weighted volumes for HGGs and FLAIR volumes for LGGs. Gross total resection was performed in 30 patients (58.8%). Preoperative FAT involvement was observed in the superior segment in 13 patients (25.5%), in the middle segment in 15 patients (29.4%), and in the inferior segment in 9 patients (17.6%). Intraoperatively, the FAT was resected in the superior segment in 11 patients (21.6%), the middle segment in 11 patients (21.6%), and the inferior segment in 6 patients (11.8%). The mean volume of FAT resected was 0.5 cm^3^ (SD 0.9). With regard to co-involvement of neighboring language-eloquent tracts, surgical intersection of the inferior fronto-occipital fasciculus (IFOF) was observed in 13 patients (25.5%), while the arcuate fasciculus (AF)/superior longitudinal fasciculus III (SLF III) complex was observed in 8 patients (15.7%) (Table 1).

**Table 1:** Population characteristics.

Characteristic	Value
IDH status	
IDH-wild type	36 (70.6%)
IDH mutant	15 (29.4%)
Tumor volume (cm^3^)	
Mean (SD)	47.4 (38.9)
Range	0.9–160.0
Extent of resection (EOR)	
Gross total resection	30 (58.8%)
Subtotal resection	17 (33.3%)
Partial resection	4 (7.8%)
Preoperative FAT involvement	
Mean (SD)	0.9 (1.2)
Range	0.0–4.8
Other tracts involvement	
IFOF	13 (25.5%)
AF/SLF III	8 (15.7%)
Superior segment involvement	
No	38 (74.5%)
Yes	13 (25.5%)
Middle segment involvement	
No	36 (70.6%)
Yes	15 (29.4%)
Inferior segment involvement	
No	42 (82.4%)
Yes	9 (17.6%)
Volume of FAT resected (cm^3^)	
Mean (SD)	0.5 (0.9)
Range	0.0–3.6
Superior segment resected	
No	40 (78.4%)
Yes	11 (21.6%)
Middle segment resected	
No	40 (78.4%)
Yes	11 (21.6%)
Inferior segment resected	
No	45 (88.2%)
Yes	6 (11.8%)
AAT—preoperative	
No aphasia	44 (86.3%)
Aphasia present	7 (13.7%)
AAT—1 week postoperative	
0	22 (43.1%)
1	16 (31.4%)
2	7 (13.7%)
3	6 (11.8%)
AAT—3 months postoperative	
0	33 (64.7%)
1	9 (17.6%)
2	7 (13.7%)
3	2 (3.9%)

### 3.2 Language function and postoperative deficits

**Figure 3:**
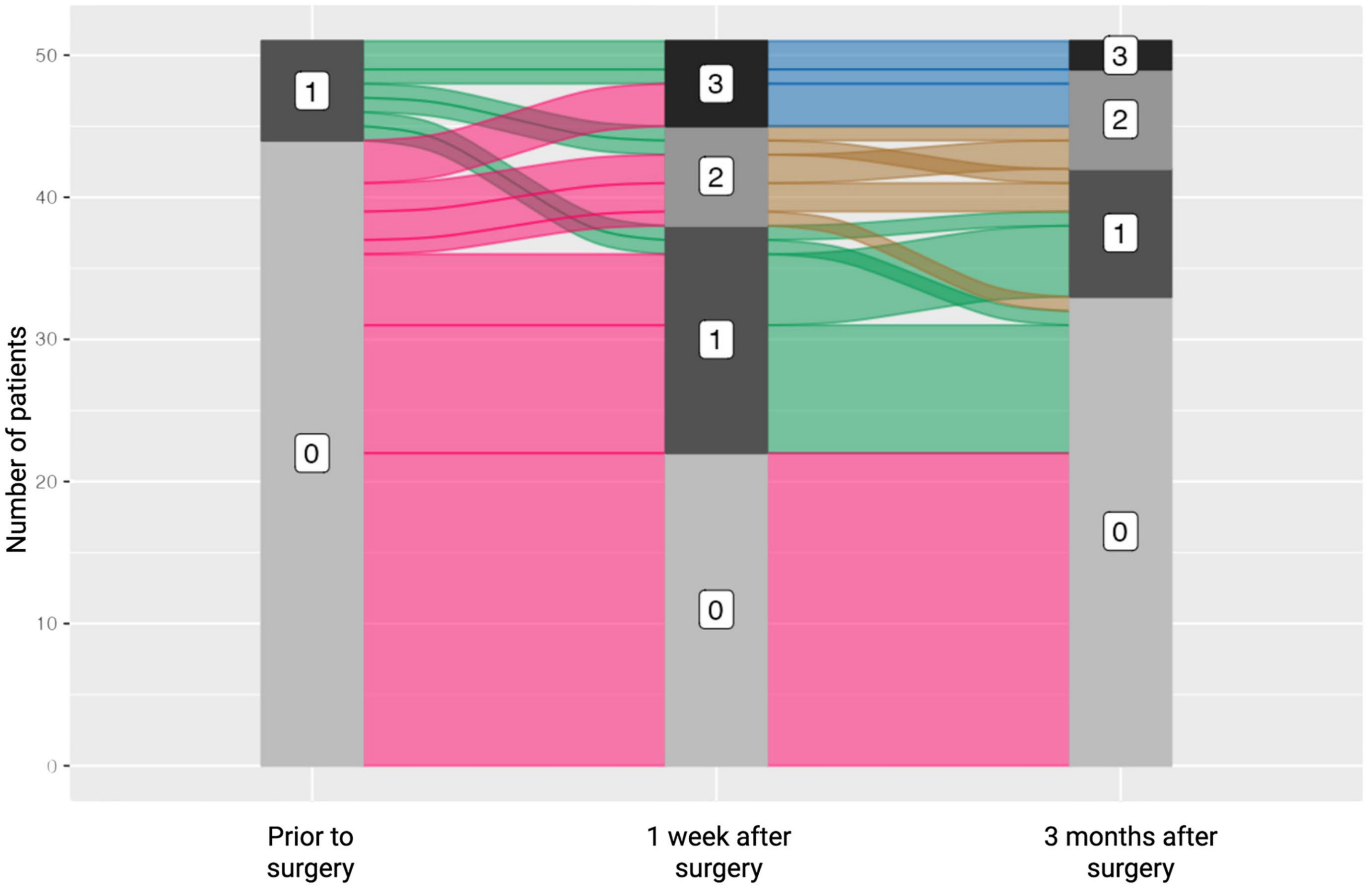
Alluvial diagram of language status preoperatively, 1 week after surgery, and 3 months later. 0 = no aphasia; 1 = mild aphasia; 2 = moderate aphasia; 3 = severe aphasia. Changing in color represents worsening of language function after surgery.

Preoperative aphasia was present in 7 patients (13.7%). At 1 week postoperatively, 29 patients (56.9%) exhibited language deficits, which improved to 18 patients (35.3%) with persistent language dysfunction 3 months later. A new postoperative language dysfunction of any type occurred in 27 patients (52.9%). Among those who developed a deficit, 18 patients (62.1%) showed subsequent improvement, while 11 patients (37.9%) did not show improvement (Table 1). At the 3-month follow-up, a persistent language deficit was present in 14 patients (27.5%) (Figure 3).

### 3.3 FAT and postoperative language outcomes

Patients with preoperative aphasia had larger tumor volumes (median 59 vs. 32.5 cm^3^, *p* = 0.05), and the inferior segment showed a more significant trend compared with the other segments (OR 4.75, 95% CI 0.85–26.7). The univariate analysis (Table 2) revealed significant associations between the development of a language deficit and several FAT-related variables: involvement of the middle segment (*p* = 0.016, OR = 5.60, 95% CI 1.34–23.4) and middle segment resection (*p* < 0.001, OR = 34.2, 95% CI 1.88–620) were strongly associated with deficit development. Mann–Whitney *U* tests confirmed that greater preoperative FAT involvement (*p* = 0.036) and larger volume of FAT resected (*p* = 0.004) were significantly associated with deficit development among continuous variables. Preoperative tumor volume (*p* = 0.385) and the extent of resection (*p* = 0.274) showed no significant association.

In patients who developed a postoperative deficit (*n* = 29), recovery was significantly associated with middle segment involvement and resection. Patients whose deficit improved had significantly lower rates of preoperative middle segment involvement (*p* = 0.018, OR = 0.11, 95% CI 0.02–0.61) and were less likely to have undergone middle segment resection (*p* = 0.048, OR = 0.16, 95% CI 0.03–0.86). Interestingly, a larger preoperative tumor volume was associated with a greater likelihood of improvement (*p* = 0.043). Preoperative FAT involvement (*p* = 0.087) and the volume of FAT resected (*p* = 0.387) were not significantly associated with improvement.

Finally, considering the analysis of persistent language deficit at 3 months, middle segment involvement (*p* < 0.001, OR = 68.0, 95% CI 10.1–458), middle segment resection (*p* < 0.001, OR = 246, 95% CI 11.8–5,131), superior segment involvement (*p* = 0.027, OR = 5.17, 95% CI 1.32–20.2), and superior segment resection (*p* = 0.005, OR = 8.25, 95% CI 1.89–36.0) demonstrated highly significant associations. Mann–Whitney *U* tests confirmed that patients with persistent deficits had significantly greater preoperative FAT involvement (*p* < 0.001) and a larger volume of FAT resected (*p* < 0.001), with a trend toward larger tumor volume (*p* = 0.059).

**Table 2:** Univariate analysis.

Variable	Deficit development (overall = 51)				Deficit improvement (overall = 29)				Persistent deficit (overall = 51)			
	No (*n* = 24)	Yes (*n* = 27)	OR (95% CI)	*p*	No (*n* = 11)	Yes (*n* = 18)	OR (95% CI)	*p*	No (*n* = 37)	Yes (*n* = 14)	OR (95% CI)	*p*
EOR				0.274				0.856				1.000
GTR	17	13			4	9			22	8		
STR	6	11			6	7			12	5		
PR	1	3			1	2			3	1		
Superior segment involvement			1.60 (0.44–5.79)	0.534			1.60 (0.44–5.79)	0.671			5.17 (1.32–20.2)	**0.027**
No	19	19			9	12			31	7		
Yes	5	8			2	6			6	7		
Middle segment involvement			5.60 (1.34–23.4)	**0.016**			0.11 (0.02–0.61)	**0.018**			68.0 (10.1–458)	**<0.001**
No	21	15			3	14			34	2		
Yes	3	12			8	4			3	12		
Inferior segment involvement			2.00 (0.44–9.07)	0.473			1.03 (0.19–5.51)	1.000			1.41 (0.30–6.62)	0.692
No	21	21			8	13			31	11		
Yes	3	6			3	5			6			
Superior segment resected			1.75 (0.44–6.93)	0.508			1.73 (0.27–11.0)	0.677			8.25 (1.89–36.0)	**0.005**
No	20	20			9	13			33	7		
Yes	4	7			2	5			4	7		
Middle segment resected			34.2 (1.88–620)	**<0.001**			0.16 (0.03–0.86)	**0.048**			246 (11.8–5,131)ᵃ	**<0.001**
No	24	16			4	14			37	3		
Yes	0	11			7	4			0	11		
Inferior segment resected			1.91 (0.32–11.5)	0.671			2.86 (0.28–29.6)	0.622			1.38 (0.22–8.50)	0.661
No	22	23			10	14			33	12		
Yes	2	4			1	4			4	2		
Preoperative language deficit (AAT > 0)			2.50 (0.44–14.3)	0.425			0.35 (0.06–2.00)	0.375			4.53 (0.87–23.7)	0.080
	22	22			7	15			34	10		
	2	5			4	3			3	4		
Preoperative tumor volume (cm^3^)	24	27	−7.0 (−22.7 to 7.0)	0.385	11	18	21.8 (1.0 to 54.0)	0.043	37	14	−17.0 (−41.8 to 0.6)	0.059
Preoperative FAT involvement (cm^3^)	24	27	−0.3 (−1.2 to −2.73e−5)	**0.036**	11	18	0.8 (−4.05e−5 to 1.6)	0.087	37	14	−1.5 (−2.1 to −0.8)	**<0.001**
FAT resected (cm^3^)	24	27	−0.5 (−0.7 to −2.66e−5)	**0.004**	11	18	0.2 (−0.5 to 0.8)	0.387	37	14	−1.2 (−1.2 to −0.7)	**<0.001**

EOR, extent of resection; GTR, gross total resection; PR, partial resection; SD, standard deviation; STR, subtotal resection. Bold values: statistically significant (*p* < 0.05).

### 3.4 Logistic regression for persistent deficit

Separate univariate logistic regression models (Table 3) quantified the individual predictive value of key variables. Preoperative FAT involvement was a significant predictor (OR = 2.38, 95% CI 1.31–4.33, *p* = 0.004) with modest explanatory power (R^2^McF = 0.176). The volume of FAT resected showed stronger predictive capacity (OR = 10.42, 95% CI 2.46–44.09, *p* = 0.001, R^2^McF = 0.380). Middle segment involvement showed the strongest univariate association (OR = 68.00, 95% CI 10.11–457.55, *p* < 0.001, R^2^McF = 0.492). Superior segment resection was also a significant predictor (OR = 8.25, 95% CI 1.89–36.05, *p* = 0.005, R^2^McF = 0.141). Middle segment resection could not be reliably estimated using a univariate logistic regression analysis due to complete separation, as no patient without a persistent deficit underwent middle segment resection (0/37 versus 11/14, Fisher’s exact *p* < 0.001).

**Table 3:** Univariate logistic regression model and multivariate logistic regression model.

Variable	Univariate analysis		Multivariate analysis	
	OR (95% CI)	*p*-value	OR (95% CI)	*p*-value
Preoperative FAT involvement (cm^3^)	2.38 (1.31–4.33)	**0.004**	0.50 (0.11–2.19)	0.358
FAT resected (cm^3^)	10.42 (2.46–44.09)	**0.001**	19.71 (1.15–338.51)	**0.040**
Middle segment involvement (yes vs. no)	68.00 (10.11–457.55)	**<0.001**	87.33 (6.05–1261.45)	**0.001**
Superior segment resected (yes vs. no)	8.25 (1.89–36.05)	**0.005**	0.28 (0.01–6.53)	0.429
Middle segment resected (yes vs. no)	3.88e+9 (0.00–Inf)	0.995	–	–

The middle segment resected was excluded from the multivariate analysis due to complete separation (zero events in the no-persistent-deficit group). Model fit: *χ*^2^ = 39.3, *p* < 0.001, R^2^McF = 0.655, R^2^N 0.777, AUC = 0.973. At 0.5 cutoff: accuracy: 92.2, 85.7%, specificity: 94.6%. Bold values: statistically significant (*p* < 0.05).

A multivariate logistic regression model was performed to identify independent predictors of persistent language deficit (Table 3). The model included preoperative FAT involvement, volume of FAT resected, middle segment involvement, and superior segment resection as predictors. The overall model was highly significant (*χ*^2^ = 39.3, *p* < 0.001) and showed excellent explanatory power (R^2^McF = 0.655, R^2^N = 0.777). Within the multivariate model, three variables emerged as significant independent predictors:

- Middle segment involvement remained a powerful predictor (OR = 87.33, 95% CI 6.05–1261.45, *p* = 0.001), indicating that preoperative tumor infiltration of this critical segment independently increased the odds of persistent deficit by approximately 90-fold.
- The volume of FAT resected maintained its independent predictive value (OR = 19.71, 95% CI 1.15–338.51, *p* = 0.040), confirming a dose-dependent relationship between the extent of tract resection and lasting language impairment.
- Preoperative FAT involvement (*p* = 0.358) and superior segment resection (*p* = 0.429) were not significant independent predictors when adjusting for other variables.

Multicollinearity diagnostics were acceptable, with variance inflation factors (VIF) ranging from 1.36 to 3.93.

**Figure 4:**
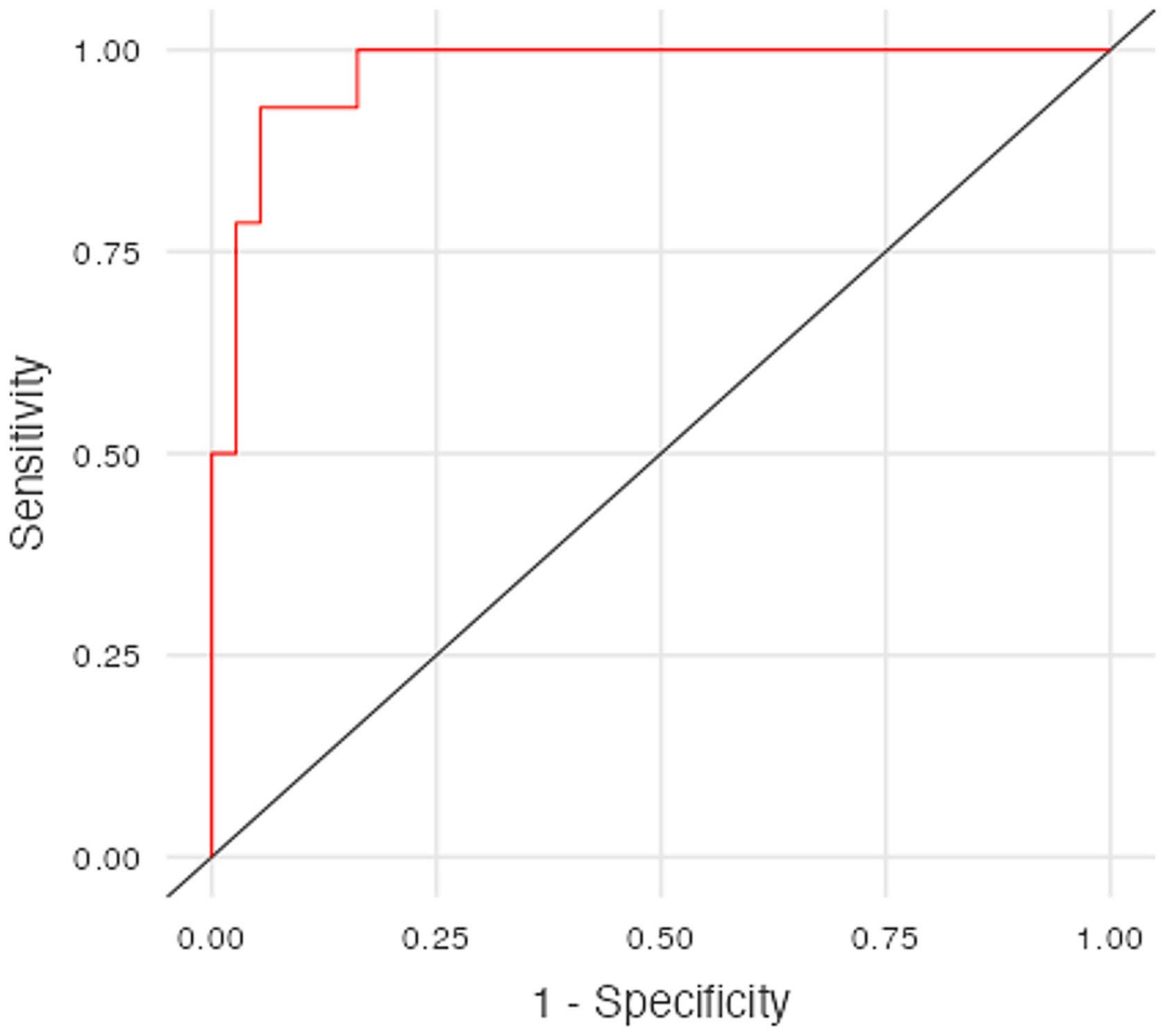
Receiver operating characteristic (ROC) curve for logistic regression-based models analyzing relations of resected FAT volume to the functional outcome.

The multivariate model showed outstanding discriminative ability, with an area under the receiver operating characteristic (ROC) curve (AUC) of 0.973, indicating near-perfect capacity to distinguish patients with persistent language deficits from those without persistent language deficits. At a probability cutoff of 0.5, the model correctly classified 92.2% of cases, with a sensitivity of 85.7% (correctly identifying 12 of 14 patients with persistent deficit) and a specificity of 94.6% (correctly identifying 35 of 37 patients without persistent deficit) (Figure 4). This high predictive accuracy supports the potential clinical utility of these FAT-related variables for preoperative risk stratification and surgical planning.

## 4 Discussion

This multicenter study investigated the role of the FAT in language function following left frontal glioma surgery. Our findings confirm that the FAT is a critical hub for language production and suggest that, to minimize the risk of long-term dysfunction, surgical strategy should prioritize preservation of its middle segment and limit the extent of resection whenever feasible.

The FAT, connecting the superior frontal gyrus (SFG) with the pars triangularis and pars opercularis of the inferior frontal gyrus (IFG) and the anterior insula (13), participates in multiple aspects of language processing, eventually contributing to verbal fluency and lexical decision-making, supporting the integration of grammatical rules in sentence production, and playing a role in speech motor inhibition (23). Additional functions related to the FAT include orofacial movements, working memory, social communication, and music processing (14). Whereas the right FAT is associated with visuospatial integration within manual/limb and oculomotor systems, the left FAT is believed to have a specific role in verbal planning and control of the articulatory apparatus (24).

In this cohort of patients, comparing those presenting with mild aphasia preoperatively (13.7%) and those without language disturbances, the degree of FAT involvement did not differ significantly. The volume of the tumor itself showed a trend toward association with the deficit, even if not statistically significant. When analyzing the segment of the FAT related to the tumor, a positive trend was described between preoperative mild aphasia and the involvement of the inferior segment, although this could also be explained by the intersection of other white matter tracts or cortical areas deputed to language function, such as the pars triangularis or opercularis of the IFG. Although FAT status was not a significant predictor of immediate postoperative language function, a reduced likelihood of deficit was observed in patients with a greater preoperative FAT involvement, suggesting a potential neural plasticity mechanism, particularly in slowly growing tumors.

The role of the FAT in language production could be better understood by analyzing intraoperative data or surgery-related deficits. In fact, this tract is integrated within the negative motor network: intraoperative electrical stimulation of the FAT can induce speech arrest or delayed speech initiation, and its disruption has been associated with postoperative stuttering (25, 26). In our experience, although GTR could be obtained only in 58.8% of cases, 56.9% of patients woke up after surgery with some degree of aphasia. A longitudinal language assessment 1 week after surgery showed worsening of the preoperative status, described as the emergence of a new deficit or worsening of those already existing, that was experienced in 52.9% of cases. Language outcomes appeared to be strongly associated with surgical manipulation, supporting the fact that partial or complete injury to the FAT may result in aphasia, difficulty in speech initiation, reduced verbal fluency, anomia, errors in verb generation tasks, and impairments in reading and simple calculations (27–29). Although a particular relationship with motor deficits has been described in association with damage to the posterior segment of the FAT in the acute postoperative phase (16) and, at the same time, a quick recovery after surgery-related FAT damage has been attributed to preservation of the posterior segment (15), we observed a significant association between acute worsening and resection of the middle segment of FAT. The development of postoperative language impairment is not only related to the segment of FAT resected but is also associated with the volume of the resection, which constituted the strongest independent predictor in our analysis. Data about a possible area of “safe resection” has not been described in the literature; we found that each additional cubic centimeter resected substantially increased the odds of a new deficit, and a volume of 0.5 cm^3^ was discovered as cutoff for the appearance of the deficit. However, it should be acknowledged that white matter tracts do not exist as isolated entities but represent a simplification of a continuous and overlapping connectome and that co-resection of neighboring tracts inevitably contributes to the observed functional outcomes to a degree that cannot be fully disentangled. In our cohort, surgical involvement of the IFOF was observed in 25.5% of cases and of the arcuate fasciculus/SLF III complex in 15.7%, reflecting the spatial proximity of these tracts to the FAT.

Although, as described earlier, knowledge and preservation of white matter tract fibers is of pivotal importance in glioma surgery, reducing the possibility of clinical worsening, FAT damage has been described as transient and was not associated with permanent aphasia (15). The mechanism of recovery of function after injury to the FAT is not currently understood, and no clear association has been found between the extent of FAT damage and functional recovery (30). Deficits caused by FAT lesions are often transient in nature, and recovery after FAT damage indicates redundancy of the tract’s function, recruitment of other brain regions, or plasticity of diffuse cortical neural networks (24, 31). Notably, tumor biology may also modulate language recovery potential: IDH-mutant tumors, predominant among lower-grade gliomas, are associated with slower growth and greater capacity for cortical reorganization compared with IDH-wildtype tumors such as glioblastoma, which afford less opportunity for functional neuroplastic adaptation (6, 32).

Transient postoperative language deficits are frequently reported in the literature, and the findings in this study demonstrate that FAT injury can also result in long-lasting impairment, with 14 patients (27.5%) showing persistent deficits at 3 months. The results confirmed the possibility of recovery after surgery, with 66.7% of patients who worsened after surgery showing subsequent improvement at the 3-month follow-up. Resection of the FAT—particularly involvement of its middle segment—emerged as the main determinant of a poor 3-month outcome, along with larger tumor volume, greater preoperative FAT infiltration, and larger volume of resection. Notably, no patient with an intact middle FAT developed a persistent deficit, whereas its resection was strongly associated with incomplete recovery at 3 months.

### 4.1 Limitations

Some limitations of this study need to be addressed. The relatively limited number of patients could reduce the significance of the results. The inclusion of patients who underwent surgery under general anesthesia (asleep surgery) may have increased the number of patients who experienced postoperative worsening. Regarding imaging, tractography was obtained using deterministic modeling rather than probabilistic methods, which is more susceptible to inaccuracies in areas of low fractional anisotropy or regions where fiber tracts intersect in multiple directions. Furthermore, co-resection of neighboring language-eloquent tracts represents an inherent confounder that cannot be fully disentangled from FAT-specific effects. A comparison of preoperative DTI studies and postoperative T1-weighted or FLAIR sequences without tract reconstruction could represent a bias in calculating the FAT excision area due to misalignments caused by brain shift, although the use of dynamic image fusion in the neuronavigation system and the acquisition of postoperative MRI within 24–48 h partially mitigate these effects. To understand the long-term language outcome, particularly regarding the potential for recovery, the follow-up period could be extended.

## 5 Conclusion

Tractography of language pathways can help determine the individual aphasia risk profile preoperatively and should guide the extent of resection during surgery to preserve neurological function. The FAT is a clinically relevant “hub” within the subcortical language network that should not be underestimated in surgical planning. The integrity of the middle FAT segment is critical for both postoperative language outcomes and long-term recovery.

## Data Availability

The raw data supporting the conclusions of this article will be made available by the authors, without undue reservation.

## References

[1] Ius T Isola M Budai R Pauletto G Tomasino B Fadiga L . Low-grade glioma surgery in eloquent areas: volumetric analysis of extent of resection and its impact on overall survival. A single-institution experience in 190 patients: clinical article. J Neurosurg. (2012) 117:1039–52. doi: 10.3171/2012.8.JNS12393, 23039150

[2] Bianconi A Bonada M Zeppa P Bruno F La Cava P Panico F . Double fluorescence-guided surgery with 5-ALA and fluorescein sodium in grade 2 and grade 3 adult-type diffuse gliomas: retrospective analysis of 112 cases. Brain Spine. (2025) 5:104277. doi: 10.1016/J.BAS.2025.104277, 40487874 PMC12145845

[3] Karschnia P Young JS Dono A Häni L Sciortino T Bruno F . Prognostic validation of a new classification system for extent of resection in glioblastoma: a report of the RANO resect group. Neuro-Oncology. (2022) 25:940–54. doi: 10.1093/NEUONC/NOAC193, 35961053 PMC10158281

[4] De Witt Hamer PC Robles SG Zwinderman AH Duffau H Berger MS. Impact of intraoperative stimulation brain mapping on glioma surgery outcome: a meta-analysis. J Clin Oncol. (2012) 30:2559–65. doi: 10.1200/JCO.2011.38.4818, 22529254

[5] Sanai N Berger MS. Operative techniques for gliomas and the value of extent of resection. Neurotherapeutics. (2009) 6:478–86. doi: 10.1016/J.NURT.2009.04.005, 19560738 PMC5084184

[6] Armocida D Bianconi A Zancana G Jiang T Pesce A Tartara F . DTI fiber-tracking parameters adjacent to gliomas: the role of tract irregularity value in operative planning, resection, and outcome. J Neuro-Oncol. (2025) 171:241–52. doi: 10.1007/S11060-024-04848-3, 39404938 PMC11685273

[7] Cepeda S Romero R Luque L García-Pérez D Blasco G Luppino LT . Deep learning-based postoperative glioblastoma segmentation and extent of resection evaluation: development, external validation, and model comparison. Neurooncol Adv. (2024) 6. doi: 10.1093/NOAJNL/VDAE199, 39659831 PMC11631186

[8] Henderson F Abdullah KG Verma R Brem S. Tractography and the connectome in neurosurgical treatment of gliomas: the premise, the progress, and the potential. Neurosurg Focus. (2020) 48:E6. doi: 10.3171/2019.11.FOCUS19785, 32006950 PMC7831974

[9] Duffau H Gatignol P Mandonnet E Capelle L Taillandier L. Intraoperative subcortical stimulation mapping of language pathways in a consecutive series of 115 patients with grade II glioma in the left dominant hemisphere. J Neurosurg. (2008) 109:461–71. doi: 10.3171/JNS/2008/109/9/0461, 18759577

[10] Mandelli ML Caverzasi E Binney RJ Henry ML Lobach I Block N . Frontal white matter tracts sustaining speech production in primary progressive aphasia. J Neurosci. (2014) 34:9754–67. doi: 10.1523/JNEUROSCI.3464-13.2014, 25031413 PMC4099550

[11] Broce I Bernal B Altman N Tremblay P Dick AS. Fiber tracking of the frontal aslant tract and subcomponents of the arcuate fasciculus in 5-8-year-olds: relation to speech and language function. Brain Lang. (2015) 149:66–76. doi: 10.1016/J.BANDL.2015.06.006, 26186231

[12] Salvati LF De Marco R Palmieri G Minardi M Massara A Pesaresi A . The relevant role of navigated Tractography in speech eloquent area glioma surgery: single center experience. Brain Sci. (2021) 11. doi: 10.3390/BRAINSCI11111436, 34827434 PMC8616013

[13] Briggs RG Allan PG Poologaindran A Dadario NB Young IM Ahsan SA . The frontal aslant tract and supplementary motor area syndrome: moving towards a Connectomic initiation Axis. Cancers (Basel). (2021) 13:1–13. doi: 10.3390/CANCERS13051116, 33807749 PMC7961364

[14] La Corte E Eldahaby D Greco E Aquino D Bertolini G Levi V . The frontal aslant tract: a systematic review for neurosurgical applications. Front Neurol. (2021) 12. doi: 10.3389/FNEUR.2021.641586, 33732210 PMC7959833

[15] Young JS Morshed RA Mansoori Z Cha S Berger MS. Disruption of frontal aslant tract is not associated with long-term postoperative language deficits. World Neurosurg. (2020) 133:192–5. doi: 10.1016/J.WNEU.2019.09.128, 31574328

[16] Nakajima R Kinoshita M Okita H Shinohara H Nakada M. Disconnection of posterior part of the frontal aslant tract causes acute phase motor functional deficit. Brain Cogn. (2021) 151:105752. doi: 10.1016/J.BANDC.2021.105752, 33993006

[17] Prasse G Meyer HJ Scherlach C Maybaum J Hoffmann A Kasper J . Preoperative language tract integrity is a limiting factor in recovery from aphasia after glioma surgery. Neuroimage Clin. (2023) 37:103310. doi: 10.1016/J.NICL.2022.103310, 36586359 PMC9817026

[18] Herbet G Maheu M Costi E Lafargue G Duffau H. Mapping neuroplastic potential in brain-damaged patients. Brain. (2016) 139:829–44. doi: 10.1093/brain/awv394, 26912646

[19] Tuncer MS Salvati LF Grittner U Hardt J Schilling R Bährend I . Towards a tractography-based risk stratification model for language area associated gliomas. Neuroimage Clin. (2021) 29:102541. doi: 10.1016/j.nicl.2020.102541, 33401138 PMC7785953

[20] Fekonja L Wang Z Bährend I Rosenstock T Rösler J Wallmeroth L . Manual for clinical language tractography. Acta Neurochir. (2019) 161:1125–37. doi: 10.1007/S00701-019-03899-0, 31004240 PMC6525736

[21] Louis DN Perry A Wesseling P Brat DJ Cree IA Figarella-Branger D . The 2021 WHO classification of tumors of the central nervous system: a summary. Neuro-Oncology. (2021) 23:1231–51. doi: 10.1093/neuonc/noab106, 34185076 PMC8328013

[22] Luzzatti C De Bleser R Scola I Frustaci M Willmes K. Update on the psychometric properties for the Italian version of the Aachen aphasia test (IT-AAT). Aphasiology. (2023) 37:658–95. doi: 10.1080/02687038.2022.2037501

[23] Cipolotti L Molenberghs P Dominguez J Smith N Smirni D Xu T . Fluency and rule breaking behavior in the frontal cortex. Neuropsychologia. (2020) 137:107308. doi: 10.1016/J.NEUROPSYCHOLOGIA.2019.107308, 31866432 PMC6996283

[24] Dick AS Garic D Graziano P Tremblay P. The frontal aslant tract (FAT) and its role in speech, language and executive function. Cortex. (2018) 111:148–63. doi: 10.1016/J.CORTEX.2018.10.015, 30481666 PMC6461388

[25] Yokoyama R Enatsu R Kanno A Suzuki H Suzuki Y Sasagawa A . Negative motor networks: electric cortical stimulation and diffusion tensor imaging. Rev Neurol (Paris). (2020) 176:592–600. doi: 10.1016/J.NEUROL.2019.12.005, 32147203

[26] Neef NE Anwander A Bütfering C Schmidt-Samoa C Friederici AD Paulus W . Structural connectivity of right frontal hyperactive areas scales with stuttering severity. Brain. (2018) 141:191–204. doi: 10.1093/BRAIN/AWX316, 29228195 PMC5837552

[27] Ille S Engel L Kelm A Meyer B Krieg SM. Language-eloquent white matter pathway Tractography and the course of language function in glioma patients. Front Oncol. (2018) 8. doi: 10.3389/FONC.2018.00572, 30574455 PMC6291459

[28] Kinoshita M de Champfleur NM Deverdun J Moritz-Gasser S Herbet G Duffau H. Role of fronto-striatal tract and frontal aslant tract in movement and speech: an axonal mapping study. Brain Struct Funct. (2015) 220:3399–412. doi: 10.1007/S00429-014-0863-0, 25086832

[29] Sierpowska J Gabarrós A Fernandez-Coello A Camins A Castañer S Juncadella M . Morphological derivation overflow as a result of disruption of the left frontal aslant white matter tract. Brain Lang. (2015) 142:54–64. doi: 10.1016/J.BANDL.2015.01.005, 25658634

[30] Agyemang K Rose A El SM Asha M Molinari E Fullerton NE . Two cases of SMA syndrome after neurosurgical injury to the frontal aslant tract. Acta Neurochir. (2023) 165:2473–8. doi: 10.1007/S00701-022-05466-6, 36625909 PMC10477090

[31] Garic D Broce I Graziano P Mattfeld A Dick AS. Laterality of the frontal aslant tract (FAT) explains externalizing behaviors through its association with executive function. Dev Sci. (2019) 22:e12744. doi: 10.1111/DESC.12744, 30159951 PMC9828516

[32] Altieri R Bianconi A Caneva S Cirillo G Cofano F Corvino S . Quantitative evaluation of neuroradiological and morphometric alteration of inferior Fronto-occipital fascicle across different brain tumor histotypes: an Italian multicentric study. Acta Neurochir. (2025) 167:71. doi: 10.1007/S00701-025-06488-6, 40072663 PMC11903521

